# Primary Adrenal Lymphoma

**DOI:** 10.4274/tjh.2012.0125

**Published:** 2014-06-10

**Authors:** Karima Kacem, Sami Zriba, Raihane Ben Lakhal, Walid Bouteraa, Lamia Aissaoui, Ramzi Ben Amor, Yosr Ben Abdennebi, Zaher Belhadj Ali, Hela Ben Abid, Balkis Meddeb

**Affiliations:** 1 Tunis El Manar University Faculty of Medicine, Aziza Othmana Hospital, Department of Hematology, Tunis, Tunisia; 2 Tunis El Manar University Faculty of Medicine, Military Hospital, Department of Internal Medicine, Tunis, Tunisia

**Keywords:** Lymphoma, Adrenal glands, Addisonian crisi

## Abstract

Primary non-Hodgkin’s lymphoma of the adrenal gland is rare. We report the case of a 56-year-old patient suffering from B symptoms. The CT scan showed a bilateral adrenal mass without any lymph nodes. Scan-guided biopsies led to the diagnosis of diffuse large B-cell lymphoma. The medullar biopsy eliminated a secondary lymphoma. The patient was treated by immunochemotherapy with a complete response before autologous stem cell transplantation.

## Abstract (tr)

Adrenal bezin Hodgkin dışı lenfoması nadirdir. Burada B semptomları ile başvuran 56 yaşında bir olgu bildirilmektedir. Bilgisayarlı tomografide bilateral adrenal kütle saptandı. Eşlik eden patolojik büyüklükte lenfadenopati görülmedi. Görüntüleme eşliğinde yapılan biyopsiler ile diffüz büyük B hücreli lenfoma tanısı kondu. Kemik iliği biyopsisi sekonder lenfoma tanısını dışladı. Hastada immunokemoterapi uygulaması ile otolog kök hücre nakli öncesinde tam yanıt sağlandı.

## INTRODUCTION

Primary adrenal lymphoma (PAL) is a rare extranodal non-Hodgkin’s lymphoma and seems to have very poor prognosis.

## INTRODUCTION

Primary adrenal lymphoma (PAL) is a rare extranodal non-Hodgkin’s lymphoma and seems to have very poor prognosis.

## CASE PRESENTATION

A 56-year-old man presented with a 2-month history of fever, night sweats, abdominal pains, and weight loss.

Clinical examination found a body temperature of 37 °C and normal blood pressure, heart rate, and respiratory rate. There was no superficial lymphadenopathy.

Laboratory findings showed a normal blood cell count, normal serum sodium, potassium of 5.82 mg/L, normal phosphorus, calcium of 2.82 mmol/L, and creatinine of 117.5 µmol/L. Blood urea nitrogen was 13.75 mmol/L. Lactate dehydrogenase was 5210 UI/L (10 times the normal upper limit).

**Figure 1:**
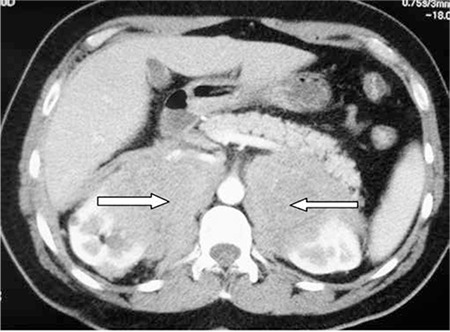
Postcontrast CT scan showed bilateral adrenal tumors (⇒).

An ultrasound scan disclosed a retroperitoneal tumor. Abdominal computed tomography (CT) scanning demonstrated bilateral adrenal tumors measuring 145x90 mm without any lymphadenopathy (Figure 1).

Positron emission tomography with 18F-fluorodeoxyglucose (FDG-PET) was not available in our country. Informed consent was obtained.

**Figure 2:**
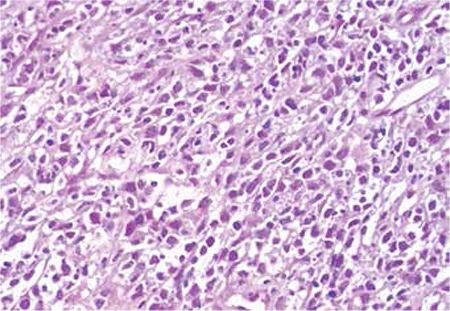
DLBCL: malignant lymphoid cells, hematoxylin & eosin stain.

**Figure 3:**
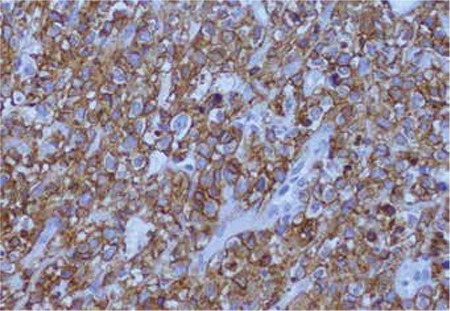
DLBCL: malignant lymphoid cells, CD20 immunostain.

Histological examination of the percutaneous CT-guided biopsy specimen from the adrenal tumor revealed CD20-positive diffuse large B-cell non-Hodgkin’s lymphoma (DLBCL) (Figures 2 and 3). 

The bone marrow biopsy specimen was normal. Chest CT scan was normal. 

Primary bilateral adrenal lymphoma was diagnosed. The disease was staged as IV according to the Ann Arbor system, and it was high-grade according to the International Prognostic Index (IPI). In accordance with Tunisian protocol, this young patient (<60 years) received a CHOP-like regimen followed by autologous stem cell transplantation (ASCT). The patient was treated by 6 cycles of immunochemotherapy type R-CHOP14 (a combination of cyclophosphamide at 750 mg/m2 on day 1, vincristine at 2 mg on day 1, adriamycin at 50 mg/m2 on day 1, and oral prednisolone at 60 mg/m2 daily for 5 days) with anti-CD20 at 375 mg/m2 on day 1. He also received intrathecal chemotherapy (methotrexate, 15 mg and hydrocortisone, 15 mg) for central nervous system prophylaxis on day 1 of the 4 cycles of chemotherapy. 

A complete endocrine work-up was performed before R-CHOP14 and showed normal plasma adrenocorticotropic hormone at 08:00 hours of 42 ng/L, normal serum cortisol at 08:00 hours of 693 mmol/L, low serum aldosterone of 10 pg/mL, low dehydroepiandrosterone sulfate of 15.4 µg/mL, and elevated serum renin of 26 ng/L. 

The patient started adrenal hormone replacement with glucocorticoid and mineralocorticoid therapy before initiating the R-CHOP chemotherapy regimen.

The clinical outcome after 4 cycles of R-CHOP was poor, with hematologic toxicities (grade IV of the WHO scale), malnutrition, adrenal crisis, Karnofsky index of 30%, and muscular atrophy. He required metabolic, nutritional resuscitation and motor rehabilitation for 1.5 months.

**Figure 4:**
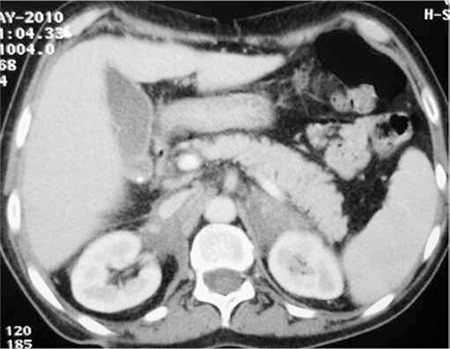
CT scan after 4 cycles of chemotherapy showed partial response.

An abdominal CT scan after the fourth cycle of chemotherapy showed a 55% regression of the adrenal tumor (Figure 4). Mobilization was done with an R-CHOP regimen and granulocyte colony-stimulating factor following the fifth cycle with collection of peripheral stem cells of 3.90x106 CD34/kg.

**Figure 5:**
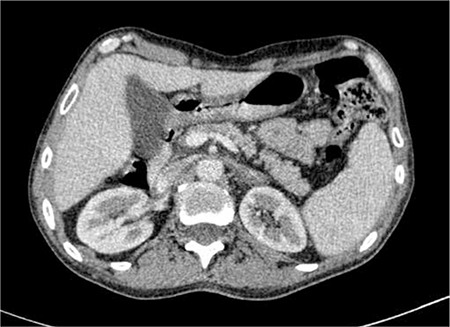
CT scan at the end of treatment showed a complete response.

The follow-up abdominal scan after 6 cycles of chemotherapy revealed no evidence of tumor (Figure 5). 

The atient received ASCT after a BEAM conditioning regimen. The patient is still alive in complete response 24 months after ASCT. 

## DISCUSSION

PAL is a rare extranodal non-Hodgkin’s lymphoma [1]. Malignant lymphoma arising in the endocrine glands represents only 3% of extranodal malignant lymphomas and is usually confined to the thyroid gland [2,3]. PAL occurs predominantly in males, with a male to female ratio of about 7:1 [3]. Our patient was a male subject. The average age is 70 years old (range=39-87) according to the existing literature [4,5]. 

These lymphomas are usually present with large, bilateral adrenal masses with or without lymphadenopathy [3]. Between 50% and 70% of patients with bilateral PAL have clinical or biochemical evidence of primary adrenal insufficiency (PAI) [5]. Since Addisonian crises have led to severe life-threatening consequences, immediate substitution therapy must be considered if PAI is suspected. PAI was found in our patient with clinical symptoms: fatigue, anorexia, weight loss, and pigmentation of skin, in addition to laboratory findings. PAI is due to infiltration and complete destruction of the adrenal glands by lymphoid cells. However, Dutta et al. suggested that there was no correlation between the size of the tumor and PAI. The bilateral adrenal lymphoma could develop because of lymphatic tissues existing in both adrenal glands [3]. The etiology of PAL is not clear; the most common hypothesis involves hematopoietic tissue akin to adrenal myelolipoma resting in the adrenal glands [6]. Ellis and Read [7] thought that the adrenal glands in humans do not contain lymphoid tissue and the follicle center cell origin of PAL suggests that the tumor may have arisen on a background of previous autoimmune adrenalitis consistent with the finding of primary adrenal insufficiency. Bilateral enlargement of the adrenal glands should raise the suspicion of lymphoma [5,6,7,8]. 

A diagnosis of PAL is made in the case of a patient presenting with bilateral adrenal masses without nodal involvement and absence of involvement of other organs [5]. PAL is usually discovered in postmortem examination [7,8,9]. PAL usually presents with bilateral adrenal masses (73%) with variable enlargement sizes ranging from 4 to 17 cm2. Density on CT scan is variable, and on magnetic resonance imaging lymphoma was described to be of low signal intensity on T1 and high signal intensity with areas of mixed signal intensity on T2 weighted imaging [5]. PAL should be differentiated from tuberculosis, nonfunctioning adenoma, pheochromocytoma, adrenal carcinoma, and metastases of the lung, breast, kidney, pancreas, and melanomas [8]. PAL is called an “incidentaloma” because it is surprisingly found on radiological investigations conducted with increasing rates for abdominal pain [9,10]. The diagnosis of PAL is confirmed by histology study. Surgical biopsy or ultrasound- or CT-guided core biopsy is required. DLBCL is the most common type of PAL (70%), followed by mixed large and small cell, small noncleaved cell, and the undifferentiated type. On immunostaining, the majority of this entity is seen to have originated from B cells [8,9,10,11]. 

At presentation, the involvement of other sites outside the adrenals is rare, but during the course of the disease PAL exhibits a propensity for generalized involvement of multiple extranodal regions such as the liver, stomach, and central nervous system. Different modalities are used for the treatment of PAL: surgery with bilateral adrenalectomy, multiagent chemotherapy, radiotherapy, or a combination of these. For PAI, replacement therapy is necessary. PAL seems to have very poor prognosis; overall, 34% of the treated patients survived disease-free for at least 6 months. Because of limited data and the limited follow-up period in most case reports, it is not possible to calculate overall survival and disease-free survival. 

Our patient is in complete response at 24 months after ASCT. ASCT was recommended in upfront consolidation therapy in our protocol for aggressive lymphoma with an IPI score of ≥2 in young patients. This decision was based on the results of study LNH87-2 of the Adult Lymphoma Study Group (French: Groupe d’Etudes des Lymphomes de l’Adulte) before the rituximab era [12]. In the literature, about 70 cases were published with rare complete or partial responses, with an overall survival of 4 months [10,11,12,13]. Kim et al. reported recent data about 14 PAL patients treated with an R-CHOP regimen. Results were encouraging and they demonstrated that achieving complete response after R-CHOP is predictive of survival. They also proposed a modified staging system for PAL in which early stages (I, II) significantly correlate with longer overall survival and progression-free survival [14]. 

At present, it is unclear whether the adjunction of rituximab to CHOP can cure patients with high-risk, aggressive lymphoma without ASCT. In our patient, it is further unknown whether prolonged complete response was due to R-CHOP alone or to the additional ASCT. It is necessary to emphasize that early diagnosis of PAL, before adrenal insufficiency appears, contributes to decreasing the patient’s morbidity and mortality.

## CONFLICT OF INTEREST STATEMENT

The authors of this paper have no conflicts of interest, including specific financial interests, relationships, and/or affiliations relevant to the subject matter or materials included.
